# Single Atoms as Growth Directors: From Graphene Edges to Atomically Precise Interfaces in 2D Materials

**DOI:** 10.1021/acsnano.6c08237

**Published:** 2026-07-01

**Authors:** Alicja Bachmatiuk, Mark H. Rümmeli

**Affiliations:** † Electron Beam Emergent Additive Manufacturing (EBEAM) Centre, Centre for Nanotechnology (CNT), Centre for Energy and Environmental Technologies (CEET), VSB-Technical University of Ostrava, 17. Listopadu 15, Ostrava 70800, Czech Republic; ‡ Institute for Materials Chemistry, IFW Dresden, 20 Helmholtz Strasse, Dresden 01069, Germany; § Faculty of Chemistry, Wroclaw University of Science and Technology, Wybrzeze Wyspiarskiego 27, Wroclaw 50-370, Poland; ∥ Key Laboratory of Advanced Carbon Materials and Wearable Energy Technologies of Jiangsu Province, Key Laboratory of Core Technology of High Specific Energy Battery and Key Materials for Petroleum and Chemical Industry, Soochow Institute for Energy and Materials Innovation, College of Energy, Soochow University, Suzhou 215006, China; ⊥ School of Smart Materials and Future Energy, Anhui Normal University, Wuhu 214002, China

**Keywords:** single-atom catalyst, crystal growth, edge-directed growth, nucleation control, 2D heterostructure

## Abstract

Single-atom catalysts are often framed as isolated reactive sites that maximize atom efficiency in chemical transformations. A less explored role is their function as growth directors, viz., atomic-scale agents that bias nucleation pathways, steer incorporation events, and shape early-stage morphologies with precision beyond that of nanoparticles. The strongest experimental evidence comes from graphene, where advanced scanning tunneling and scanning/transmission electron microscopies enable direct tracking of atoms at growth edges and kinks, linking configurations to stepwise growth. First-principles studies on Rh(111) propose that transition-metal single atoms, particularly Mo, can promote productive feeding species such as diatomic carbon and boron nitride (BN) dimers, lower kinetic barriers during early h-BN−graphene lateral heterostructure growth, and influence boundary chemistry. This perspective reframes single atoms as growth directors, distills the mechanistic insights established for graphene, extends them to emerging heterostructures, and outlines criteria for identifying single-atom-directed growth, providing a basis for the rational design of atomically precise 2D interfaces.

## Graphical abstract

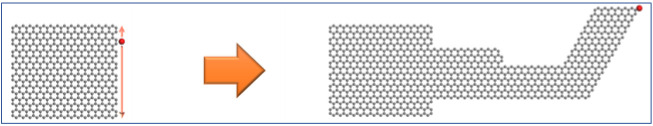

Single-atom catalysts are typically discussed in the context of chemical transformations, where their primary role is to maximize atom efficiency and provide well-defined active sites. In this context, the isolated atom is treated as a reactive center that accelerates or redirects specific reactions. However, when the process of interest is crystal growth rather than chemical conversion, the functional role of a single atom must be reconsidered. At this scale, an isolated atom can act not only as a catalytic site but also as a growth-directing element that locally modifies the energy landscape, stabilizes specific feeding units, and mediates incorporation at the growth front. For the purposes of this perspective, a single-atom catalyst (SAC) refers to an isolated atom that remains atomically dispersed under the relevant growth conditions. More specifically, we define SAC-directed growth as growth processes in which such an isolated atom actively participates at the growth front and influences nucleation, incorporation, or boundary formation events. This definition distinguishes SAC-directed growth from related concepts such as supported single-atom catalysts, where isolated atoms primarily function as catalytic active sites, and single-atom alloys, where isolated guest atoms modify the properties of a host surface. While these systems may influence growth indirectly, the focus here is on isolated atoms that directly participate in and direct the growth process itself. In addition, single-atom alloys represent a related but distinct concept. In these systems, isolated guest atoms are dispersed within a host metal surface and can modify catalytic activity through local changes in adsorption and reaction energetics. Such effects may influence crystal growth indirectly; however, the present perspective focuses on isolated atoms that directly participate in growth-front processes, including nucleation, incorporation, and boundary formation. Consequently, single-atom alloys are considered adjacent to, but generally outside, the primary scope of SAC-directed growth discussed here. This distinction is particularly relevant for low-dimensional materials, where structural features such as edges, kinks, and atomic terminations govern electronic and mechanical properties. In these systems, catalyst nanoparticles typically operate at length scales significantly larger than the lattice features they influence, limiting their ability to enforce deterministic atomic structures. By contrast, an isolated atom can, in principle, interact with the lattice at the same scale as the growth event itself, enabling localized control over nucleation, boundary formation, and defect evolution in low-dimensional materials.

The clearest experimental demonstrations of this concept arise from studies of graphene, where atomic-scale imaging and simulations have enabled direct tracking of individual atoms at active growth sites. These results provide a mechanistic foundation for defining single-atom catalytic growth as a distinct growth mode. While Yang et al.^1^ used graphene as a benchmark system to establish the foundations of single-atom catalytic growth, the present Perspective extends the discussion by framing isolated atoms as growth directors, broadening the scope toward atomically precise interfaces in two-dimensional materials, and proposing practical criteria for identifying genuine single-atom-directed growth across material systems.

## Single-Atom Catalytic Growth as a Distinct Growth Mode

A central message emerging from the graphene-focused single-atom catalytic (SAC) growth literature is scale matching: when the functional properties depend on edge bonding, atomic termination, defect-local structures, or atomically precise interfaces, a catalyst particle is often too large to enforce deterministic atomic boundaries, whereas an isolated atom can, in principle, operate at the same length scale as the lattice. This distinction is not limited to nanoparticle catalysts. Conventional metal catalyst surfaces and thin-film alloy catalysts can provide highly active environments for crystal growth; however, their influence is typically distributed across many surface atoms and adsorption sites, making it difficult to directly associate specific incorporation events with individual atomic centers. By contrast, an isolated atom offers the possibility of atomically localized control, where feeding units, incorporation barriers, and boundary formation may be modified at a specific growth-active site. From this perspective, a single-atom catalyst is not merely highly efficient; it is also a growth director that can create local energy minima, stabilize transient building blocks, and mediate events that involve the incorporation of one atom (or one minimal feeding unit) at a time. At the same time, SAC-directed growth introduces additional challenges, including maintaining single-atom stability and experimentally verifying direct participation at the growth front.^1^

This distinction motivates the need for an operational definition. Single-atom catalytic growth is most convincingly demonstrated in cases in which the isolated atom (i) is identified as such and (ii) participates at the growth front during the incorporation steps, rather than in cases where single atoms are present elsewhere on the surface without demonstrated involvement in the growth.^1^ Practically, this definition is demanding, which explains why relatively few convincing examples have been reported. The limited progress likely reflects the demanding requirements for high spatiotemporal resolution and mechanistic control, which often require a combination of in situ microscopy and computation.^1^

Graphene has become the benchmark platform for SAC-directed growth for two reasons. First, its edges (zigzag, armchair, and reconstructed forms) provide discrete, chemically meaningful growth fronts, whose terminations strongly influence the properties and device concepts.^1^ Second, the atomic contrast and stability of graphene enable the direct observation of single atoms at or near growth fronts within certain experimental windows. As summarized by Yang et al.,^1^ two complementary regimes supported growth (scanning tunneling microscopy (STM) + theory) and freestanding growth (transmission electron microscopy (TEM) + theory), converge on a consistent mechanistic motif: single atoms preferentially localize at growth-active geometries (kinks, steps, edge discontinuities), where they raise the likelihood of productive incorporation by (i) increasing the residence time at the active site and (ii) promoting sequential, row-by-row edge advance rather than sporadic attachment/detachment events. An important consideration is that the effectiveness of a growth-directing atom depends not only on its presence at a growth-active site but also on the balance between diffusion and incorporation processes. As these kinetics are strongly system-dependent, future studies should consider atom mobility within the specific growth environment when evaluating SAC-directed growth mechanisms.

Density functional theory (DFT)-driven analyses of graphene growth via chemical vapor deposition (CVD) predict that single adatoms can stabilize specific edge structures and lower incorporation barriers. Shu et al. showed that Cu passivation at the armchair edges is energetically preferred and can substantially lower the incorporation barriers, thus providing a kinetic rationale for edge-selective growth outcomes.^2^ Wang et al. extended this logic, indicating that single Ni adatoms can mediate insertion at kink sites, precisely the type of local geometry for which a single-atom *tool* could control row completion.^3^ The supported-growth case is particularly compelling because it enables direct real-time observation. Patera et al. imaged epitaxial graphene growth on Ni(111) using high-speed STM and observed that individual Ni atoms trapped at kink sites were acting as nucleation centers; the growth proceeded through the orderly completion of interrupted carbon rows as the kink advanced^4^ (Figure 1, panel a). Yang et al. further emphasized that molecular dynamics simulations revealed Ni adatoms diffusing on the metal surface and then along the edges of graphene, with prolonged residence at kinks, consistent with a catalytic role at these sites.^1,4^ Together, these results establish a supported-growth *micromechanism* that is highly transferable as a concept even when the material system changes: (i) single-atom capture at a geometrically defined growth site; (ii) edge-parallel diffusion; (iii) residence-time enhancement at kinks; (iv) feeding-unit attachment near the atom; (v) row completion and kink propagation.^1–4^

Freestanding growth provides the most direct visual evidence because individual atoms can be tracked and followed as they advance at the open edges. Zhao et al. reported direct in situ observations of single-Fe-atom catalytic processes at graphene edges by linking single-atom motion with incorporation events and anomalous diffusion behaviors.^7^ A subsequent report discussed analogous growth behavior for single Cr atoms at the graphene edges^5^ (Figure 1, panel b). These studies have strongly shaped the field because they establish the growth-director concept as a repeatable cycle rather than a static picture of the active site. Two additional lines of evidence sharpen the boundary between the growth and competing processes. Kano et al. showed that isolated Cu atoms can mediate *mending/healing* of graphene edges to provide a mechanistically distinct but related outcome: single atoms can catalyze bond rearrangements and edge reconstruction routes that restore crystalline order.^8^ Second, Ramasse et al. provided direct evidence of suspended graphene being etched by a metal-mediated process,^9^ reinforcing the key message from the review that growth and etching can be closely competing channels, and interpreting *single-atom activity* requires careful control of the carbon chemical potential and experimental conditions.^1,9^

A particularly important nuance in the graphene literature is the role of the experimental window. Electron beam environments can provide not only imaging but also energy input and feedstock availability through hydrocarbon contamination. Rümmeli et al. discussed the electron-beam-driven chemistry in and around graphene^10^ and reported room-temperature electron-beam-driven graphene growth from hydrocarbon contamination using TEM.^11^ These two investigations are valuable for two reasons: they provide practical routes to observe atomic-scale events, but they also act as reminders that electron-beam-driven regimes are a distinct growth environment, and conclusions about general synthesis must be carefully framed.^1,10,11^

## Beyond Graphene: What Generalization Should Look Like

A recurring conclusion of this graphene-focused perspective is that, beyond graphene, convincing demonstrations of growth driven strictly by a single atom remain sparse, largely because of the difficulty in simultaneously satisfying (i) single-atom identification, (ii) growth-front localization, (iii) mechanistic controls, and (iv) causal linkages to growth outcomes.^1^ Rather than treating this as a limitation, it can be leveraged as a roadmap; generalization should proceed by transferring mechanistic logic (feeding units, barrier reduction, residence-time effects, and boundary selectivity) to other material systems while maintaining clear validation criteria. The mechanistic concepts emerging from graphene also suggest possible future opportunities in other classes of two-dimensional materials, although convincing demonstrations of SAC-directed growth remain limited. Transition-metal dichalcogenides (TMDs), including MoS_2_−WS_2_ lateral heterostructures, are particularly attractive candidates because their properties depend strongly on interface sharpness, edge structure, and boundary chemistry. In principle, isolated atoms located at growth fronts could influence feeding-unit stabilization, incorporation barriers, and boundary selectivity in a manner analogous to that observed for graphene. However, direct experimental evidence remains sparse, and establishing causal links between isolated atoms and growth outcomes represents an important future challenge.

Similar opportunities may exist in MXenes and other surface-terminated two-dimensional materials, where termination chemistry and nucleation pathways strongly influence structural evolution and functional properties. Although SAC-directed growth has not yet been demonstrated in these systems, atomic-scale growth directors could potentially provide new routes for controlling termination selectivity, interface formation, and defect evolution. More broadly, these examples highlight how the graphene benchmark may serve as a conceptual framework for exploring SAC-directed growth across a wider range of low-dimensional materials. Table 1 summarizes representative opportunities, challenges, and current evidence levels for extending SAC-directed growth beyond graphene-based systems.

**Table 1:** Representative Opportunities, Challenges, and Current Evidence Levels for Extending Single-Atom Catalytic (SAC)-Directed Growth Beyond Graphene-Based Systems^a^

Material Class	Representative Systems	Growth Challenge	Potential SAC Role	Current Evidence Level
Graphene	Graphene, graphene nanoribbons	Edge propagation, defect control, atomic termination	Growth-front localization, feeding-unit stabilization, row-by-row incorporation	Strong experimental and theoretical evidence
h-BN−Graphene Heterostructures	Lateral h-BN/graphene interfaces	Interface formation, boundary chemistry, nucleation control	Stabilization of C_2_ and BN feeding units, modulation of incorporation barriers, interface engineering	Theory-supported with emerging mechanistic framework
Transition Metal Dichalcogenides (TMDs)	MoS_2_−WS_2_, MoSe_2_−WSe_2_ lateral heterostructures	Interface sharpness, edge-selective growth, boundary control	Barrier modulation, feeding-unit stabilization, control of interface formation	Conceptually promising; direct evidence currently limited
MXenes and Surface-Terminated 2D Materials	Ti_3_C_2_T_*x*_ and related MXenes	Termination control, nucleation selectivity, defect evolution	Stabilization of preferred growth pathways, termination-selective growth, interface control	Conceptual opportunity; no direct demonstrations reported
Other Emerging 2D Materials	2D oxides, metallenes, elemental 2D materials	Nucleation control, phase selection, interface engineering	Growth-front stabilization, phase-selective incorporation, atomic-scale boundary control	Future opportunity

a While convincing demonstrations remain largely confined to graphene the mechanistic principles of feeding unit stabilization barrier modulation , residence-time enhancement and boundary selectivity suggest broader applicability across range of two-dimensional materials.

Given the limited experimental evidence currently available beyond graphene, theoretical investigations play an especially important role in identifying promising material systems, mechanistic pathways, and experimentally testable design principles for SAC-directed growth. In this context, theoretical studies become particularly valuable when they go beyond reporting adsorption energies and instead provide specific kinetic pathways, feeding-block concepts, and design metrics that can be experimentally tested. Zhu et al. provide a timely example of theory-led generalization, targeting the catalytic growth of atomically thin hexagonal boron nitride (h-BN)−graphene lateral heterostructures on Rh(111) by depositing transition-metal single atoms (TM = Mn, Zr, Nb, Mo, Hf, Ta, W).^6^ Their central claim is explicit and mechanistic: On Rh(111), where C−C dimerization is unfavorable on the pristine surface, transition metal (TM) single atoms, particularly Mo, act as SACs that (i) promote C adatom dimerization both thermodynamically and kinetically and (ii) stabilize BN dimers, thereby accelerating the early nucleation and migration steps required for rapid lateral heterostructural growth.^6^ They further proposed that Mo can dynamically participate in and migrate out of the interface while carrying C_2_ as a feeding block, and can tune the boundary that links C−N to C−B, thus giving rise to distinct electronic properties^6^ (Figure 1, panel c).

**Figure 1:**
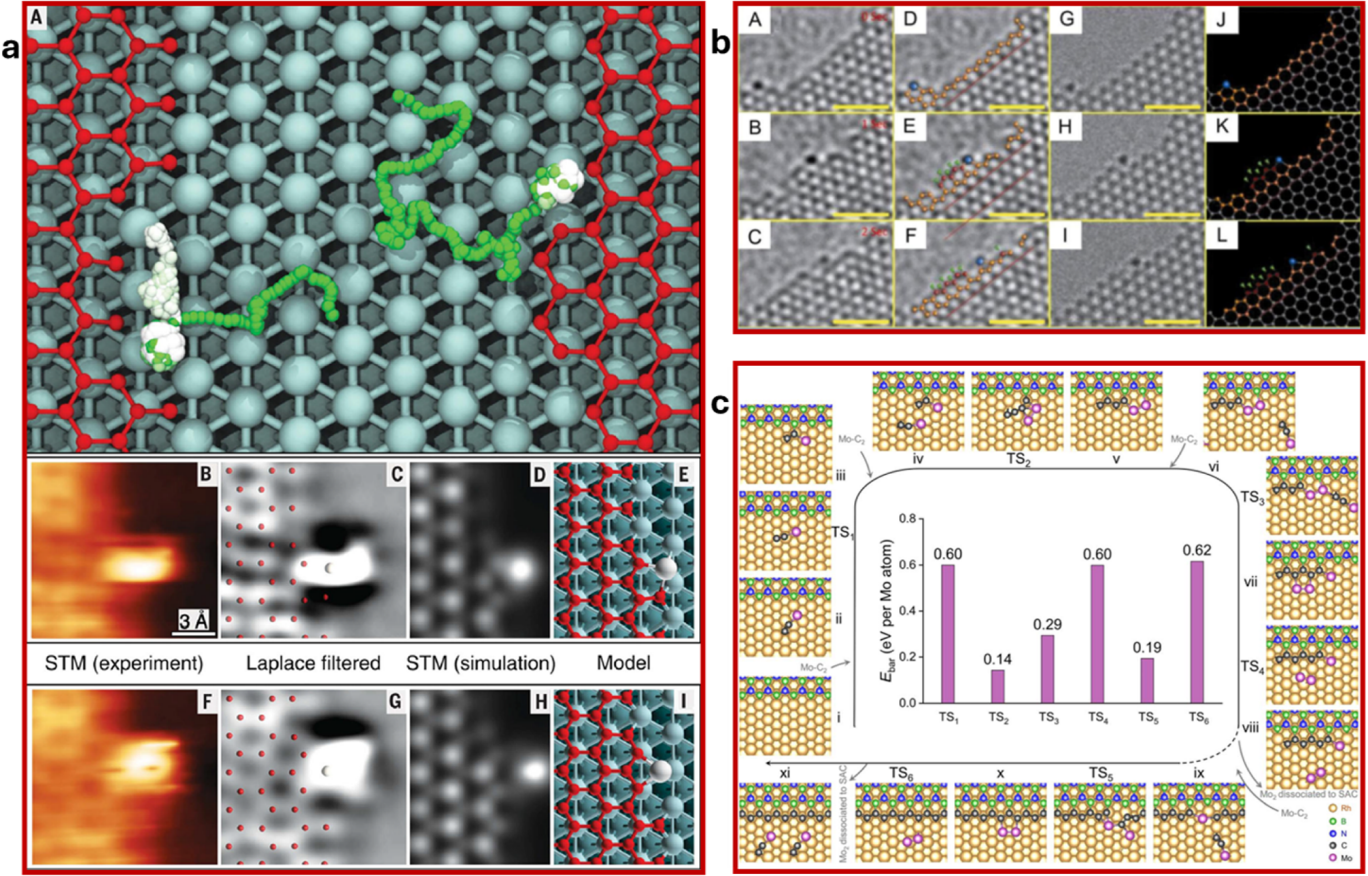
Panel a: Nickel adatoms at the graphene edges. A Ni adatom diffusing on a surface in a region delimited by graphene z (right) and k (left) edges with kinks. Two representative trajectories obtained by molecular dynamics simulations with ReaxFF performed at 710 K for 100 ps are shown. Color palette for Ni trajectories: from green (initial position) to white (final position). The final steps are highlighted by increasing the ball size. B−I Short-lived configurations of Ni adatom at k-edge kinks: B and F High-speed STM images from movie S2 (20) (*V* = 20 mV; *I* = 7 nA). C and G Laplace-filtered version of the images in (B) and (F) with superimposed ball models. D and H constant-height STM simulated images based on the calculated geometries (E and I). Adapted with permission from Patera, L. L., et al., *Science* 2018, 359, 1243−1246. Copyright 2018 American Association for the Advancement of Science. Panel b: Catalytic growth of graphene by a single Cr atom at the graphene edge under electron beam irradiation. A−C, with partial stick-and-ball models to aid viewing (D)−(F), image simulations of the growth process G−I, and complete stick-and-ball models J−L. The blue ball indicates Cr, whereas red balls and green arrows signify new C atoms. All scale bars are 1 nm. Adapted with permission from Ta, H. Q., et al., Nano Research 2018, 11, 2405−2411. Copyright 2018 Springer Nature. Panel c: The MEP for the initial nucleation stage in forming 1D h-BN-G. Here, the Mo−C_2_ configurations serve as the seeds attaching to the zigzag B edge of the h-BN domain. The values of *E*_bar_ per Mo atom for the key steps (from TS1 to TS6) are shown in the illustration. Adapted with permission from Zhu, Y., et al., *J. Mater. Chem. A* 2024, 12, 30498−30507. Copyright 2024 Royal Society of Chemistry.

Two features of this work deserve emphasis from a *growth director* perspective:

### Feeding Blocks Are Central, Not Incidental

Instead of focusing solely on monomeric attachment, Zhu et al. treated C_2_ and BN dimers as *productive growth units*. They computed the minimum energy pathways and barriers for key attachment steps, which they then connected to the kinetic picture (including Arrhenius-type rate comparisons at representative temperatures).^6^ For BN supply, they showed that the attachment of a BN dimer to the graphene edge has a lower barrier than separate B or N monomers; in addition, with the assistance of Mo-SAC, the barrier can be lowered further, which supports the *SAC stabilizes the right building block* motif.^6^

### Barrier Reduction Is Tied to a Specific Bonding Mechanism

Their crystal orbital Hamilton population (COHP) and integrated COHP (ICOHP) analyses provide a quantitative bonding interpretation: Mo-SAC weakens C−Rh coupling while enhancing C−C bonding in the relevant configurations, which they argue explains the more facile migration/dimerization and lower kinetic barriers.^6^ This maps onto the general design logic emerging from the graphene benchmark: reducing overbinding to the substrate while stabilizing productive incorporation units, thereby reshaping the early stage energy landscape. Zhu et al. discussed scenarios involving Mo_2_ dimers and their dissociation into single Mo atoms, with explicit pathways described in the Supporting Information.^12^ From a single-atom growth perspective, this does not automatically negate the single-atom hypothesis. Instead, it highlights a practical requirement for future experiments: claims must be anchored to the active state during the decisive steps (and dynamic speciation should be measured/controlled rather than assumed). The availability of a specific dissociation pathway (Mo_2_ → Mo) and diffusion barriers is exactly the type of mechanistic accounting that should become standard when extending SAC-growth beyond graphene.^12^ More broadly, extending these concepts beyond the current benchmark systems raises important questions regarding the universality of SAC-directed growth mechanisms across different growth environments. An additional challenge for future studies is determining how SAC-directed growth mechanisms translate from metal-supported systems to weakly interacting substrates such as SiO_2_, sapphire, or hBN, which are widely used for the growth and integration of two-dimensional materials. In such environments, substrate-mediated catalytic effects may be reduced, potentially increasing the relative importance of localized growth-front interactions and feeding-unit stabilization. At present, direct experimental evidence remains limited. Future investigations may therefore benefit from model platforms that combine atomically resolved characterization with controlled precursor delivery, including graphene-supported TEM experiments, graphene-coated TEM grids with engineered feedstocks, and advanced in situ microscopy approaches capable of tracking isolated atoms under realistic growth conditions. Exploring SAC-directed growth on weakly interacting substrates remains an important challenge for extending the framework beyond current benchmark systems.

A clear set of operational criteria is useful for both authors and reviewers to maintain a distinction between single-atom and cluster-mediated growth. Drawing directly from the graphene benchmark and its interpretational challenges,^1–5,7–11^ the following four criteria provide a minimal system-agnostic *proof package*:

### Criterion 1: Single-Atom Identity

The catalytic species must be identified as an isolated atom (element + coordination environment where possible), ideally with statistics (counting, distributions).^1,4,5,7,8^

### Criterion 2: Persistence under Growth Conditions

The isolated atom must be shown to remain isolated under the conditions in which growth occurs (electron dose and temperature dependence, as needed), which explicitly excludes hidden subnanometer clusters.^1,10,11^ Because atom stability depends strongly on the substrate, temperature, chemical environment, and irradiation conditions, universal stability windows are unlikely to exist. Consequently, persistence should be evaluated within the specific growth environment under investigation using appropriate experimental and computational controls.

### Criterion 3: Growth-Front Participation

The atom must be located at the step/edge/kink/interface where incorporation occurs; proximity alone is insufficient.^4,5,7^

### Criterion 4: Causality

The growth rate, directionality, or boundary chemistry must change in response to the introducing/removing/poisoning of the atom or modifying its binding geometry, ideally with controls.^2–4,6,9^

These criteria are intentionally demanding because the term single-atom catalytic growth is most useful when supported by rigorous experimental validation. The literature on graphene shows why identically looking experimental windows can yield growth, healing, etching, or beam-driven artifacts depending on the feedstock and energy input.^1,9–11^

## Concluding Perspective

Beyond summarizing the current state of the field, this Perspective proposes a conceptual framework in which isolated atoms are viewed as growth directors rather than solely catalytic active sites. By combining mechanistic insights from graphene with emerging examples of interface-controlled growth, we outline a pathway for extending SAC-directed growth concepts across a broader range of two-dimensional materials and provide practical criteria for evaluating future claims of single-atom-directed growth. The graphene benchmark demonstrates that single atoms can act as growth directors through geometry-specific trapping at kinks and edges, residence-time enhancement, and stepwise incorporation cycles, as observed using STM and (S)­TEM.^1–5,7,8^ It also underscores key interpretational challenges: growth must be distinguished from etching and electron-beam-driven carbon supply (when relevant), and the active state must be verified rather than inferred.^9–11^ The Rh(111) theory provides a credible path toward generalization by explicitly formulating a kinetic growth mechanism in which transition-metal single atoms stabilize productive feeding blocks (C_2_, BN dimers), lower nucleation/migration barriers, and even tune the chemistry at the boundaries of lateral heterostructures.^6,12^ The most productive next step for this research field is therefore not only to expand the range of materials, but also to refine mechanistic descriptors and validation criteria that enable controlled, atomically precise growth and design of 2D interfaces beyond graphene.

## Abbreviations

BN: boron nitride
COHP: crystal orbital Hamilton population
CVD: chemical vapor deposition
DFT: density functional theory
ICOHP: integrated crystal orbital Hamilton population
SAC: single-atom catalytic
STM: scanning tunneling microscopy
TEM: transmission electron microscopy
TM: transition metal
